# NClassG+: A classifier for non-classically secreted Gram-positive bacterial proteins

**DOI:** 10.1186/1471-2105-12-21

**Published:** 2011-01-14

**Authors:** Daniel Restrepo-Montoya, Camilo Pino, Luis F Nino, Manuel E Patarroyo, Manuel A Patarroyo

**Affiliations:** 1 Intelligent Systems Research Laboratory - LISI, Universidad Nacional de Colombia, Carrera 45 No. 26-85, Bogotá DC, Colombia; 2 Research Group on Combinatorial Algorithms - ALGOS-UN, Universidad Nacional de Colombia, Bogotá DC, Colombia; 3 School of Medicine and Health Sciences, Universidad del Rosario, Carrera 24 No. 63C-69, Bogotá DC, Colombia; 4 School of Medicine, Universidad Nacional de Colombia, Bogotá DC, Colombia; 5 Fundación Instituto de Inmunología de Colombia - FIDIC, Carrera 50 No. 26-20 Bogotá DC, Colombia

## Abstract

**Background:**

Most predictive methods currently available for the identification of protein secretion mechanisms have focused on classically secreted proteins. In fact, only two methods have been reported for predicting non-classically secreted proteins of Gram-positive bacteria. This study describes the implementation of a sequence-based classifier, denoted as NClassG+, for identifying non-classically secreted Gram-positive bacterial proteins.

**Results:**

Several feature-based classifiers were trained using different sequence transformation vectors (frequencies, dipeptides, physicochemical factors and PSSM) and Support Vector Machines (SVMs) with Linear, Polynomial and Gaussian kernel functions. Nested *k*-fold cross-validation (CV) was applied to select the best models, using the inner CV loop to tune the model parameters and the outer CV group to compute the error. The parameters and Kernel functions and the combinations between all possible feature vectors were optimized using grid search.

**Conclusions:**

The final model was tested against an independent set not previously seen by the model, obtaining better predictive performance compared to SecretomeP V2.0 and SecretPV2.0 for the identification of non-classically secreted proteins. NClassG+ is freely available on the web at http://www.biolisi.unal.edu.co/web-servers/nclassgpositive/

## Background

Machine Learning (ML) tools have been successfully applied to the solution of a variety of biological problems such as the classification of proteins according to their subcellular localization and secretion mechanism. Different computational methods have been used to obtain reliable subcellular localization predictions, such as Artificial Neural Networks (ANNs), Hidden Markov Models (HMMs) and Support Vector Machines (SVM) [1-4].

The simplest way of addressing classification problems is to follow a binary approach, trying to discriminate objects according to two categories: positive (+) and negative (-). SVMs rely on two concepts in order to solve this type of problems: the first one is known as the large-margin separation principle, which is motivated by the idea of classifying points in two dimensions; and the second one is known as Kernel methods [5].

The Kernel methods that have been applied to bioinformatics are classified into three categories mainly: Kernels for real-valued data, Kernels for sequences and Kernels developed for specific purposes such as the Position-Specific Scoring Matrix (PSSM)-Kernel [6]. In the first case, examples that represent a data set can be usually expressed as feature vectors of a given dimensionality. In the case of Kernel functions for real-valued data, linear, polynomial and Gaussian Kernels are some of the most commonly used functions and they were used in the implementation of NClassG+. In the third case, the most frequently used Kernels for sequences are the Spectrum Kernels describing *l*-mer content [7], positional Weighted Degree (WD) Kernels that use positional information [8] and other Kernels for sequences such as the Local Alignment Kernel [5,9].

The use of Kernels for exploring real-valued biological data such as proteins usually involves two steps. In the first step, amino acid sequences are transformed into fixed-length vectors that are then used to feed ML tools so that they can learn to make predictions in a second step [10,11]. The SVM classification method outstands among the techniques based on Kernel learning, which searches for an optimal separation hyperplane in the feature space and determines the optimal data separation margin, maximizing the generalization capacity of the detected pattern. This separation hyperplane is trained by means of quadratic programming [12]. SVMs and Kernel functions are very effective for solving classification problems because they are based on probability theory, can handle large data sets of high dimensionality, and have great flexibility to model diverse data sources [5].

One of the fundamental issues of computational biology is directly associated with representing data as objects in a given space; this is of key importance for the solution of classification and clustering problems. For example, in the case of protein sequences, their variable lengths do not allow the use of vector representations [13], a problem known as the "sequence metric problem", which is directly associated with the use of an alphabetic letter code that lacks an implicit metric and, therefore, it is not suitable for making comparisons between such objects [5,14]. To solve this problem, different sequence representations have been proposed based on features and similarity measures, some of which are shown in Table 1[5,14-18].

**Table 1:** Comparison of the evaluation measurements of NClassG+, SecretomeP 2.0 and SecretP 2.0 for the classification of Gram-positive bacterial proteins

	NClassG+		SecretomeP 2.0		SecretP 2.0	
	**Split set^a3^**	**Test set**	**Split set^a3^**	**Test set**	**Split set^a3^**	**Test set**

Accuracy	0.88	0.90	0.88	0.84	0.69	0.83
MCC	0.77	0.71	0.76	0.52	0.46	0.50
Specificity	0.92	0.97	0.88	0.71	1.00	0.99
Sensitivity	0.84	0.87	0.86	0.54	0.34	0.32

The split set is a partition of the learning data set used in the training process, and the test set corresponds to the independent set used in the final test only for comparing the performance of NClassG+, SecretomeP 2.0 and SecretP 2.0. The split sets (a_3_) correspond to the ones reported in Figure 2 (B step).

Over the last 20 years the use of the ML techniques mentioned above have allowed proposing novel solutions to the identification of protein secretion and post-translational modifications. The validation of the different methods available for predicting protein secretion [19,20], as well as the use of such algorithmic methods for the identification of potential drug and vaccine target proteins, followed by the experimental validation of such predictions [21,22], have shown to be a consistent approach to obtain novel biological findings supported on computational processes and with direct application to the solution of protein secretion problems.

ML tools used in the identification of secreted proteins have been developed taking into account the biological principles of protein subcellular localization, which is essential for the correct functioning of these proteins [1]. The localization of secreted proteins in their appropriate cellular compartments involves diverse processes that range from the transport of small molecules through highly complex routes with intrinsic sequence signaling processes. Much of the current efforts in understanding protein secretion have focused on how such protein transportation systems work and on the identification of membrane proteins to drive drug development toward products that have specific effects on such proteins [23,24].

In Gram-positive bacteria, proteins might localize in at least four different locations: the cytoplasm, cytoplasm membrane, cell wall and extracellular milieu. Since protein synthesis takes place in the cytoplasm, secreted proteins have to be transported across the cell membrane so that they can fulfill their function effectively [25-27]. Given the complexity of such secretion systems, it is not surprising that new mechanisms of secretion are being constantly discovered [28]. Thus, there is a considerable number of proteins that have been experimentally identified as secreted but whose mechanism or route of secretion has not been yet identified and therefore are said to be secreted via non-classical or alternative means [29].

Many of the proteins that are secreted via alternative pathways are directly associated with pathogenic processes, thus their identification is of key importance [30]. In the case protein secretion in Gram-positive bacteria, there are six secretion systems to transport proteins across the cytoplasmic membrane reported up to date: secretion (Sec), twin-arginine translocation (Tat), flagella export apparatus (FEA), fimbrilin-protein exporter (FPE), hole forming (holing) and WXG100 secretion system [30,31]; however, it is important to emphasize that non-classical protein secretion should not be considered as a single mechanism but rather as a range of secretion systems that differ from classical secretion but are still not clearly characterized. This discloses problems both with the experimental and computational strategies currently used to identify new secretory mechanisms and highlights the importance of developing new strategies to study non-classical secretion.

The development of this work focused on the identification of non-classically secreted proteins. It is worth noting that for some of these secreted proteins a known function has been also reported in the cytoplasm, leading to their classification as "moon-lightning" or multi-functional proteins. NClassG+ identifies proteins that are secreted through signal-peptide independent pathways and was here validated based on a compiled list of extracellular proteins lacking a signal peptide. NClassG+ was compared to the two available algorithms for classifying non-classically secreted Gram-positive proteins, named SecretomeP 2.0 [29] and SecretP 2.0 [32].

## Results

A training and a split set were built from a learning data set containing 420 positive proteins and 433 negative proteins with thoroughly adjusted parameters. Independently, a test set containing 82 positive examples of non-classically secreted proteins and 263 negative examples were constructed for comparing NClassG+ to the other classifiers of non-classical secretion. These data sets were the result of removing redundant proteins with more than 25% of identity. Linear, polynomial and Gaussian Kernel functions were selected for constructing the representation vectors, as literature revision indicated that these are very well explored Kernel functions. The data sets were supported on experimental reports and the necessary vector transformations were applied to them during the learning process.

A nested *k*-fold CV procedure was used to tune the model and compute the error separately. This was done with the aim of finding the best parameters to train the complete data set. The exploration was optimized using a grid search approach and led to proposing a classifier, which was trained independently on frequencies, dipeptides, factors and PSSM vectors as well as on all possible combinations between such vectors. The predictive behavior of NClassG+ was analyzed and contrasted against SecretomeP 2.0 and SecretP 2.0 in two occasions: one with the split set during the training process and the other one with the test set during a separate testing step.

### Model selection

About 15 000 hyperparameter combinations comprising feature vectors, SVM C values, and Kernel functions and their parameters were explored to select the best classifier. The optimized exploration of combinations pointed to a linear classifier combining factors, dipeptides and PSSM vectors as the one that yielded the highest accuracy in the inner loop of the nested CV procedure. The C parameter of the classifier was equal to 64. The average accuracy of the outer folds in the nested *k*-fold cross-validation was 0.93.

### Evaluation measurements

**Figure 1:**
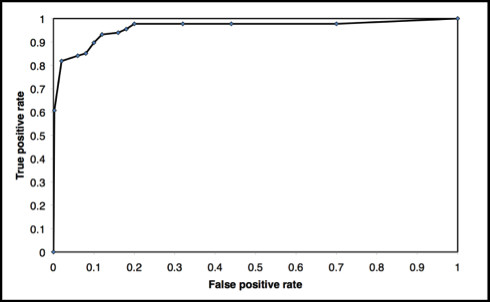
**NClassG+ ROC Plot**. ROC plot analysis of the performance of NClassG+.

In Figure 1 the ROC plot of NClassG+ shows the true positive rate (sensitivity) plotted in function of the false positive rate (specificity). The ROC plot test shows good discrimination. The graph also shows a high accuracy as the curve climbs rapidly toward the upper left hand corner of the graph.

Compared to SecretomeP and SecretP, NClassG+ showed a better performance both in the test with the split set after the training process, as well as in the independent test with the test set, as indicated by its higher accuracy and MCC. The correct identification of non-classically secreted and non-secreted proteins, understood in terms of the tools' sensitivity and specificity, were notably high for NClassG+ (both values were above 0.84), thus indicating that this tool recognizes a similar proportion of both protein types, in contrast to SecretomeP 2.0 and SecretP 2.0, in which such relationships were unbalanced (Table 1).

## Discussion

One of the most complex areas of ML is directly associated with finding and constructing training and exploration data sets [33]. In this study, a positive training set containing 3 794 protein sequences and a negative training set comprising 21 459 protein sequences were obtained by screening the SwissProt database. Both protein sets were balanced by adjusting the percentage of identity in each set.

In this study, prediction of non-classically secreted proteins is done based on a modification of classically secreted proteins, as proposed by Bendtsen and colleagues [29,34]. However, here we postulate novel training and exploration data sets that were astringently adjusted, as well as innovative data transformations and methods not previously used in the classification of non-classically secreted proteins.

It is important to highlight that the input data for the construction of NClassG+, SecretomeP 2.0 and SecretP 2.0 were all extracted from SwissProt (version 53.1 for NClassG+, version 44.1 for SecretomeP and version 57.7 for SecretP); therefore, there is probably some data overlapping between the training data sets of the three tools. Nevertheless, the diversity of protein prediction methods, the constant increase of protein data and the identification of new problems stress the importance of analyzing and extracting data to construct new hypotheses in terms of protein localization.

Different pre-processing techniques were used in the construction of the feature vectors that represented each of the sequences in the input data set. These techniques have some intrinsic computation details that can result in comparatively more expressive vectors [35]. In the specific case of dipeptide and PSSM vectors, both types of vectors use 400 features to represent each amino acid sequence, but evidently, PSSM is the vector that represents each protein more effectively. PSSM vectors have been reported to be one of the most efficient ways of representing proteins in statistical learning [16,17,36-40] but the strategy of mixing different vectors resulted in even better results in terms of the evaluation measurements.

It is worth noting that NClassG+, SecretomeP 2.0 and SecretP 2.0 use data from two biological classes of Gram-positive bacteria (Firmicutes and Actinobacteria). However, part of the features used in SecretomeP 2.0 come from prediction methods that were trained with protein sequences that belong to biological groups different from Gram-positive bacteria, which suggests that there are common secretion mechanisms among the different biological entities; however, such hypothesis should be experimentally validated in the same way as it has been done for classical secretion in Gram-positive bacteria [22,25,27,41-46].

Although both NClassG+ and SecretP 2.0 use an SVM algorithm, there are deep differences in terms of the methodology approach followed by both tools. Both tools use different techniques to build their vector representations, but SecretP 2.0 does a smaller exploration to obtain its final classifier. Yu *et al. *reported a lower ability of SecretP 2.0 to predict non-classically secreted Gram-positive proteins compared to SecretomeP 2.0 [32], which also agrees with the results of NClassG+ (Table 1). However, it is particularly interesting that SecretP 2.0 was built to classify 3 protein categories (classically secreted proteins, non-classically secreted proteins and non-secreted proteins) but was validated using classical measures (sensitivity, specificity, accuracy and MCC), which are basically adequate to evaluate binary results.

In particular for NClassG+, the linear, polynomial and Gaussian Kernel functions were explored under equal conditions for its optimization. The best results were obtained using the linear function, which is consistent with reports by Ben-Hur and colleagues [5] stating that the linear kernel provides a useful baseline and is hardly beaten in many bioinformatics applications, especially when the dimensionality of the input set is large and there is a small number of samples, as occurred with NClassG+.

In order to select the best classifier, the results were optimized according to parameters, exploring different vector combinations as well as different Kernel functions. In the case of the function exploration, it is important to mention that the Gaussian function has less difficulties compared to the polynomial function because 0 <*Kij *≤ 1, in contrast to the polynomial Kernel function, where values may tend to infinity as the degree of the polynomial increases [47]. This is observed in the nature of the variables of the polynomial function, where the number of experiments is larger compared to the other two methods (linear and Gaussian).

In the validation of the different classifiers proposed in this study, the results obtained by calculating the ROC showed good discrimination between false positives and true positive proteins. Nevertheless, it should be taken into account that the ROCs characterize the potential ranges of the algorithm but not the performance of a given classifier [48].

## Conclusions

This study reports the NClassG+ tool for the classification of Gram-positive bacterial proteins that are secreted independently of the classical secretory pathway. This tool has a novel training data set and is composed of a classifier based on a polynomial function that uses vectors built from dipeptides, frequencies and PSSM data.

Among the 4 types of vectors, the similarity-based PSSM vector was always present in the optimization process, which reflects the efficiency of this type of vector for representing protein sequences, compared to the other 3 types of vectors. However, the combination of the different vector representations was a good approach to solve the classification problem, as it minimized the optimistic biased thanks to the nested CV and allowed to obtain a robust classifier.

There are still novel protein secretion and translocation mechanisms to be discovered, where the use of computational and ML methods can play a key role for elucidating new processes and discovering new biological mechanisms.

## Methods

### Learning and test data

#### Data source

The UniprotKB (version 15.5) protein database was used as reference for constructing NClassG+ [49]. This database includes several databases such as PRI-PSD, TrEMBL and SwissProt version 53.1 [50]. Among these databases, SwissProt was used for the construction of the learning and test data sets because it is publicly available and the protein sequences reported in it have gone through a careful annotation process [51]. Until October 2009, a total of 10 424 881 proteins were reported in SwissProt; 512 994 of these proteins had been manually annotated and reviewed, while the remaining proteins were under adjustment at that time.

#### Data set selection

Proteins were selected according to the systematic classification of Gram-positive bacteria reported in SwissProt version 53.1. Accordingly, bacterial proteins are classified into two large biological classes: Actinobacteria (19 897 curated proteins reported), which are characterized by a high G+C content, and Firmicutes, which have a low G+C content [50]. As general data adjustment criteria, proteins had to be at least 50 amino acids long and no more than 10 000 amino acids in length. Sequences annotated as 'fragment', 'probable', 'probably', 'potential', 'hypothetical', 'putative', 'maybe' and 'likely', were excluded from the positive and negative sets.

#### Adjustment of the learning and test data sets

The learning (training and split sets) and the test sets (independent set) were adjusted using the PISCES algorithm [52,53]. This algorithm reduces sequence redundancies based on an identity measure by making "all against all" comparisons of PSSM matrixes obtained using PSI-BLAST (3 iterations, *E*-value: 0.0001, BLOSUM 62 matrix). Only proteins with ≤25% of identity were included within the learning and test data sets [54].

#### Learning and test data sets

The positive data set comprised only proteins whose annotation in SwissProt v.53.1 contained the words 'signal', 'secreted', 'extracellular', 'periplasmic', 'periplasm', 'plasma membrane', 'integral membrane' or 'single pass membrane'. This resulted in a set of 3 794 bacterial proteins that fulfilled all criteria. The sequence portion corresponding to the translocation mechanism (first region between position 1 up to a varying point that ranges between amino acids 21 and 55) was manually removed based on the annotation reported in SwissProt [29,34]. This procedure yielded a set of proteins that lacked a signal sequence and was only applied to this set; all other sets were not modified. The set was reduced to 420 proteins after adjusting its identity to ≤25%, as described above.

The negative protein set included proteins whose annotations contained the words 'cytoplasm' or 'cytoplasmic'. This selection criteria identified a total of 21 459 proteins. To obtain a negative set with experimental support, proteins were randomly divided into two sets. Ninety percent of the negative set was used for the learning process (training and split sets) of the classifiers and 10% of the negative set was used to complement the test data set. The first one contained 433 proteins and the second one 263 proteins after adjusting the identity to ≤25%.

For the test set (independent set), an initial screening of SwissProt v.53.1 identified 178 curated redundant proteins being secreted despite lacking a signal sequence, which formed the positive data set. Proteins labeled with the word "secreted" in the keyword line and without the word "signal" in the feature table line were selected to construct the test set, as reported by Yu *et al. *[55]; this set also included the test set reported by Bendtsen *et al. *2005 for SecretomeP. The set was depurated to 82 proteins after adjusting its identity to ≤25% and was complemented with 10% of the negative set (263 proteins) that was built based on a random partition of the redundant negative set. This set was used for analyzing the predictive capacity of NClassG+ and contrasting its predictions with the results obtained with SecretomeP 2.0 and SecretP 2.0 [29,32,34].

### Feature vectors

Protein prediction models are frequently constructed using structural and physicochemical features extracted from amino acid sequences [18]. Among the different types of data that can be used to construct feature-based vectors are amino acid composition or "frequencies" [36,56], dipeptides [57-59], physicochemical features [39], and PSSM [17].

#### Construction and normalization

Because of methodological requirements, it is necessary to transform the variable length of the protein sequences into fixed-length vectors. This step is of key importance for protein processing and classification with ML tools [40]. All the transformations explained below produce fixed-length vectors.

#### Amino acid composition vectors (frequencies)

Amino acid composition is understood as the fraction of each of the twenty amino acids in a protein sequence. With this method, proteins are described as vectors of 20 features [36,56].

#### Dipeptide vectors

These types of vectors are constructed based on the composition of dipeptides and have been extensively used to represent protein sequences [57-59]. Dipeptide composition vectors contain information regarding the frequency as well as the local order of amino acid pairs in a given sequence and describe proteins using 400 features [60,61].

#### Statistical factor vectors

On the basis of the study described by Atchley *et al. *[14], a multivariate statistical analysis was carried out over the 494 physicochemical and biological attributes predetermined for each amino acid, as it is reported in the AAindex [62]. Such study defined a set of highly interpretable factors based on the characteristics contained in this database for representing amino acid variability. These high-dimension data attributes were summarized in the following 5 factors (a) Factor I or polarity index, (b) Factor II or secondary structure factor, (c) Factor III related to the molecular size or volume with high factor coefficients for bulkiness, (d) Factor IV, which reflects relative amino acid composition, and (e) Factor V, which refers to electrostatic charge with high coefficients on isoelectric point and net charge. Based on this method, proteins are represented as vectors of 100 features [35].

#### PSSM vectors (PSI-BLAST)

Profiles of biological data with evolutive implications can be extracted using PSI-BLAST [63] to construct profiles from the estimated PSSM [17,64]. Basically, a PSI-BLAST search is carried out for each protein using the non-redundant (NR) database that contains the GenBank CDS translations, PDB, SwissProt, PIR and PRF databases, iterating thrice. PSI-BLAST parameters have to be adjusted so that the discriminating criterion of the *E*-value corresponds to 0.001, and the BLOSUM62 substitution matrix is used. This results in a PSSM from which a vector of 400 features is obtained per sequence by collapsing rows over columns, as described in detail by Jones [17]. The elements of these input vectors are subsequently divided according to the length of the sequence and are then escalated to a range between "0" and "1" using the sigmoid function [39,40,65]. This method allows constructing vectors that describe proteins using 400 features. PSSMs were locally calculated using Blastpgp [66], downloading the NR BLAST database which contains 9 993 394 protein sequences.

#### Vector processing

Amino acid composition, dipeptide composition, factors and PSSM vector combinations were explored and optimized to identify which were more expressive. The output format of the vectors corresponds to the standard output of the LIBSVM software package [67].

### Kernel methods

Taking into account the recommendations of Fan *et al. *[68] for exploring Kernel function parameters and methods, the comparison should be efficient under different conditions established by the user in order to obtain a wide approach to all the different behaviors of the classifier. Such recommendations are: (a) "Selection of parameters", which is related to performing cross-validations of the models to be trained in order to find the set of parameters that best fit the data, the Kernel function and the type of SVM, so as to obtain the final model, and (b) "Final training", which consists on training the classifiers with the complete data set based on the best set of parameters. The linear, polynomial and Gaussian Kernel functions as well as C-SVC for the SVMs were explored in the construction of NClassG+.

### Model selection

**Figure 2:**
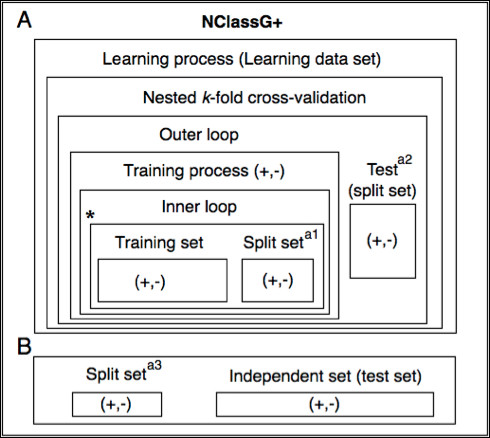
**Methodology of NClassG+**. The NClassG+ classifier was selected among a large number of possible classifiers resulting from all the possible combinations of protein vector representations and Kernel functions considered in this study. In step A, the candidate classifiers were built and compared in a nested *k*-fold cross-validation (CV) environment. Briefly, using the training and test data sets from the inner loop of the nested *k*-fold CV procedure, a classifier is optimized according to CV accuracy for all the possible Kernel function/feature combination pairs, selecting the pair with the best CV accuracy value in each iteration of the outer loop. The training and test data sets from the inner loop come from the training data set of the outer loop, the test data set from the outer loop is used to calculate an estimated accuracy of the whole process. Using the hyperparameters of the best classifier trained with the inner loop CV, a classifier is trained and tested with the outer loop data sets. NClassG+ is the classifier with the best CV accuracy, as calculated in the inner loop. In step B, prior to performing the nested *k*-fold CV procedure, the learning data set was partitioned to assess and compare the performance of the selected classifier against SecretomeP 2.0 and SecretP 2.0. The a_1_, a_2_, and a_3 _data sets are totally different partitions derived from the learning set used in the construction of NClassG+. * hyperparameter optimization.

Often the cross-validation (CV) error of the chosen model is also used for evaluating the performance of the model, which leads to obtaining an overoptimistic result, since the CV error is minimized, i.e., the chosen model is biased downwards. To avoid this problem, a better way to combine the selection of the model and the performance evaluation is by using nested *k*-fold cross-validation. In an outer loop, data are repeatedly split into subsets for learning and testing. On each learning set, the model parameters that had minimal CV error are chosen. The best model is then tested with the independent test set. The results for all test sets are averaged to obtain an estimate of the generalization error [69,70]. Hyperparameter optimization is carried out by doing a parameter grid search for all the different possible combinations of vector representations, classifiers and parameters [36]. A schematic representation of this process is shown in Figure 2.

#### ROC plot analysis

The final performance of NClassG+ was calculated based on the total average of the subsets and the performance was evaluated based on their standard parameters of sensitivity, specificity and accuracy [48,68,71].

#### Sensitivity, specificity, accuracy and Matthews correlation coefficient (MCC)

The threshold parameters of prediction methods can be set dependently or independently, and each method has its own limitations. The performance of the CV and the ability of a method to predict novel sequences can be evaluated using four threshold-independent parameters: sensitivity, specificity, accuracy and MCC. These measures were defined in terms of the following values: true positives (TP), false negatives (FN), true negatives (TN) and false positives (FP), as follows:

Sensitivity corresponds to the percentage of proteins that are correctly predicted as secreted or as TP, as shown in Equation 1.

(1) $$Sensitivity(sn)=\frac{TP}{TP+FN}100$$

Specificity is defined as the percentage of non-secreted proteins that are correctly predicted, as shown in Equation 2.

(2) $$Specificity(sp)=\frac{TN}{TN+FP}100$$

Accuracy is related to the percentage of proteins that are correctly predicted as non-classically secreted or non-secreted proteins out of the total number of protein sequences, as shown in Equation 3.

(3) $$Accuracy=\frac{TP+TN}{TP+TN+FP+FN}100$$

The MCC is defined as shown in Equation 4. An MCC of "1" means that the prediction is correct, while "0" means that the prediction is incorrect.

(4) $$MCC=\frac{\left(TP*TN)-(FP*FN\right)}{\sqrt{\left(TP+FP)(TP+FN)(TN+FP)(TN+FN\right)}}$$

## Authors' contributions

DR-M wrote the manuscript, designed and validated NClassG+. DR-M carried out data analysis and interpretation supported by CP. LFN, MEP and MAP contributed to the methodological design, supervised its development and critically revised the manuscript's content. LFN and MAP supervised the research group. All authors read and approved the final version of the manuscript.
